# Removal of Calcium Hydroxide Pastes Containing N-Methyl-2-pyrrolidone, Local Anaesthesia, Glycerine, and Methylcellulose from Artificial Radicular Grooves: An *In-vitro* Study

**DOI:** 10.14744/eej.2021.35229

**Published:** 2021-12-22

**Authors:** Afzal ALI, Banu ARICIOĞLU, Hakana ARSLAN

**Affiliations:** 1. Department of Conservative Dentistry and Endodontics, Pacific Dental College and Hospital, Rajasthan, India; 2. Department of Endodontics, Faculty of Dentistry, Recep Tayyip Erdoğan University, Rize, Turkey; 3. Department of Endodontics, Faculty of Dentistry, Istanbul Medeniyet University, Istanbul, Turkey

**Keywords:** Aqueous vehicle, calcium hydroxide, intracanal medicament (ICM), NMP, viscous vehicle

## Abstract

**Objective:**

To compare the removal of calcium hydroxide (CaOH) pastes containing N-Methyl-2-pyrrolidone (NMP), lidocaine, glycerine, methylcellulose, or water from artificially created grooves.

**Methods:**

In this study, 115 human single-rooted maxillary incisors with single and straight root canals were prepared using a rotary file up to size 40/.04 and split longitudinally. A longitudinal groove was created from 2 to 5 mm from the apex and filled with CaOH combined with different vehicles. The specimens were divided among 5 experimental groups according to the vehicle as follows: distilled water, lidocaine, glycerine, methylcellulose, and NMP. The two halves were re-attached, and the canals were flushed with 10 ml of 17% EDTA for 60 seconds. The residual amount of CaOH was scored using a stereomicroscope at 8× magnification. Statistical significance was set at P<0.05.

**Results:**

The NMP-based group exhibited significantly less residual medicament compared to the distilled water (P<0.05), while there were no statistically significant differences among the methylcellulose-, lidocaine-, and glycerine-based groups and distilled water (P>0.05).

**Conclusion:**

The vehicle is an important factor in the successful removal of CaOH medicament from the root canals. Within the limitations of the present study, the NMP-based CaOH medicament exhibited better removal efficacy than the distilled water. However, the cleaning success of the methylcellulose-, lidocaine-, and glycerine-based groups was similar to that of distilled water.

**HIGHLIGHTS**

• The NMP-based CaOH medicament removed easier.
• N-Methyl-2-pyrrolidone (NMP) from the Pyrrolidones, has strong solubilization efficiency.
• Glycerine-based paste showed a similar removal efficacy as the distilled water medicament.

## Introduction

A successful outcome following endodontic treatment is attributed to the reduction or elimination of microorganisms from the root canal system. However, current mechanical preparation techniques are insufficient to achieve a completely disinfected root canal system (1). Therefore, the use of various chemical agents and intracanal medicaments has been advocated (2).

Calcium hydroxide (CaOH) paste is the preferred intracanal medicament in endodontics. It is mainly composed of powder, a vehicle, and a radioopacifier. The vehicles combined with CaOH can change CaOH paste’s physical and chemical properties by affecting antibacterial action, radiopacity, flow, ionic release, and diffusion (3). They are commonly classified as aqueous, viscous, or oily (4). Aqueous vehicles (such as water, saline, anaesthetic solution, and methylcellulose) rapidly dissociate the CaOH to ions and provide a high concentration when in contact with fluids (5). Viscous vehicles, such as glycerine, polyethene glycol, and propylene glycol, dissolve more slowly due to their molecular weight and minimise the distribution of CaOH to the tissues. Thus, the medicament can stay longer in the root canal (6). Oily vehicles (such as olive oil, silicone oil, camphor, and parachlorophenol oil) are non-water-soluble substances and provide lower solubility and diffusion than other vehicles (7).

A new vehicle from the pyrrolidone group, N-Methyl-2-pyrrolidone (NMP), was recently introduced to the endodontic field. It is a strong dissolving agent with a low-weight molecule, making it easy for the drugs to be delivered quickly (8) NMP can weaken the hydrogen-bonded structure of water and thus act as a co-solvent owing to its non-polar carbons; the solubilising ability of drugs, drug partition, and the flux of the penetrant can be increased (9). Furthermore, it was shown in a study, when ıt was taken together with bone morphogenetic protein, it enhances the activity (10). Similarly, Gjoksi et al. (11) reported that when NMP was applied systemically on the pulp-dentine complex, it induced the activity of the pulp-dentine complex and prevented alveolar bone loss.

Although there are some conflicting results in the literature about the necessity of CaOH removal, it has been well established that the CaOH residue can reduce the penetration of the sealer into the dentinal tubules of root dentine (12). Furthermore, it may change the properties of the cementum, damage hermetic obturation, and cause apical microleakage; as a result, the long-term success of the treatment may be affected negatively (13). Until now, several studies have focused on the successful removal of CaOH combined with different vehicles (13-15). However, there is limited data about comparing conventional vehicles with NMP-based vehicles in terms of the removal of CaOH from artificial grooves. Therefore, the present study aimed to compare CaOH pastes containing NMP, lidocaine, glycerine, methylcellulose, or water in terms of removal from artificially created grooves. The null hypothesis was that there would be no significant differences regarding removal efficacy between the tested materials.

## Materials and Methods

Following local ethical committee approval (PDCH/20/EC-235), 115 extracted single-rooted maxillary incisors with single and straight roots with mature apices were selected. Teeth with more than a single root canal and apical foramen, previous root canal filling, internal/external root resorption, calcification, cracks or fractures, and immature root apices were excluded from the study. Preoperative periapical radiographic images were taken in the buccolingual and mesiodistal directions to confirm the root canal anatomy. To verify straight root anatomy, buccal and proximal radiographs were analysed. The canal curvature of each tooth was measured using imaging software (ImageJ: National Institutes of Health, Bethesda, MD, USA). Straight single-rooted teeth with single canals were included. The apical diameter of each canal was established by gently binding a #K-15 (Dentsply Sirona) to the apex. Canals with a larger apical diameter were excluded. The crowns of the teeth were sectioned at 17.5 mm from the apex to standardise the working length at 17 mm. A reservoir was then created 3 mm from the canal entrance using a round bur with a diameter of 2.3 mm (Komet, Dusseldorf, Germany, 340.202.001.001.023, size 8). A single endodontist prepared the root canals, up to size 40/0.04 Neoniti (NEOLIX, Châtres-la-Forêt, France) rotary file according to the manufacturer’s instructions; they were irrigated intermittently with 2 ml of 3% sodium hypochlorite (NaOCl) Parcan, Septodont, Saint-Maur-des-Fosses, France). The samples were dried with paper points and embedded in modified Eppendorf vials (Eppendorf-Elkay, Shrewsbury, MA) using elastomeric impression material (Optosil; Heraeus Kulzer, Hanau, Germany). All the samples were numbered and then removed from the mould. Subsequently, longitudinal grooves were prepared in all samples on the buccal and lingual surfaces with a diamond disk under copious water irrigation. They were split into two halves with the chisel. At a distance from 2 to 5 mm from the apex, a longitudinal groove (3 mm length, 0.2 mm width, and 0.5 mm depth) was created on one side of each tooth to imitate the uninstrumented canal. The grooves were cleaned with a brush. Finally, the grooves were irrigated with 5 ml of 17% ethylenediaminetetraacetic acid (EDTA) Prime Dental Products Pvt. Ltd, India), followed by 5 ml of 2.5% NaOCl for 60 seconds, and finally rinsed with 5 ml distilled water; they were dried before applying the CaOH paste. The samples were randomly allocated to five experimental groups (n=17).

Group 1: CaOH powder (Prevest Denpro Limited, Jammu, India) (0.08 mg)+distilled water (0.3 ml).

Group 2: CaOH powder+lidocaine (2% lidocaine HCl with 1:80,000 epinephrine [Xicaine, ICPA Health Products] a ratio of 0.32 mg/ 0.6 ml, approximately).

Group 3: Glycerine (ApexCal; Ivoclar Vivadent AG, Bendererstrasse, Liechtenstein, 29% CaOH).

Group 4: Methylcellulose (Calcygel; Prevest Denpro Limited, Jammu, India, 45% CaOH).

Group 5: NMP (CleaniCal; Maruchi, Wonju, Korea, 30% CaOH).

All grooves were filled with CaOH paste, and the two halves were re-attached and re-positioned in their silicone moulds in Eppendorf vials. The remaining coronal portion was filled with the same CaOH using a size 35 lentulo spiral (Dentsply Maillefer, Ballaigues, Switzerland). Finally, cotton pellets were placed, and the access cavity was temporised (Cavit, 3M ESPE, Seefeld, Germany). The samples were stored for one week at 37^°^C with 100% humidity.

### Removal of CaOH

After the storage period, the temporary filling material was removed, and the master apical file instrument was adjusted to working length manually; then, the irrigation activation was performed. For the irrigation procedure, the root canals were filled with 5 ml of 17% EDTA and agitated ultrasonically with the Endosonic Blue (Maruchi, Wonju, S Korea) Ni-Ti file #30/.02 size for 30 seconds at 29.5-30.5 kHz from 2 mm short of the working length. Ultrasonic agitation was carefully executed, enabling it to vibrate freely without touching the walls. The procedure was repeated twice, and a total of 10 ml of 17% EDTA was agitated for 60 seconds. Finally, to prevent further irrigant action, the root canals were flushed with 5 ml of distilled water and dried with paper points.

### Determination of the amount of remaining CaOH

**Figure 1.**
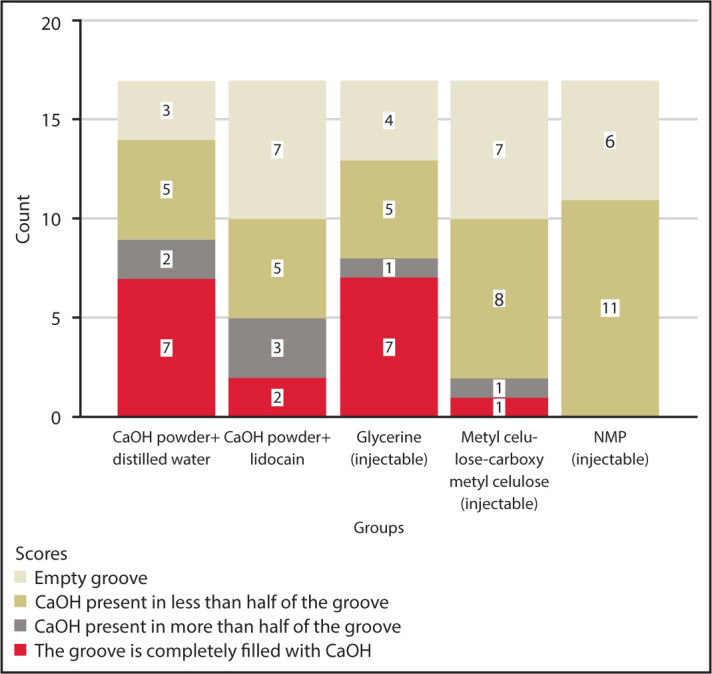
Distribution of the sample size according to the scores NMP: N-Methyl-2-pyrrolidone

The samples were taken out of the mould and disassembled into two halves to evaluate the removal of different vehicle-based CaOH. The grooves were inspected for the remaining CaOH using a stereomicroscope (Lawrence and Mayo, London) with digital images at 8× magnification. Each image was scored by two calibrated clinicians using the scoring criteria established by van der Sluis et al. (16) (Fig. 1).

Score 0=Empty cavity

Score 1=CaOH in less than half of the cavity

Score 2=CaOH in more than half of the cavity 

Score 3=Cavity filled with CaOH completely

Statistical analysis

For the analysis of the inter-examiner agreement, the kappa test was used. The reliability between the examiners was good (kappa value=0.822). The data were analysed using Kruskal-Wallis and Mann-Whitney U tests at a 95% confidence level (P<0.05). The statistical analyses were performed using IBM® SPSS® Statistics 20 software (IBM SPSS Inc., Chicago, IL, USA).

## Results

Figure 1 presents the distribution of the sample size according to the scores in each group. The NMP-based group exhibited less residual volume than the distilled water group (P<0.05). However, there were no significant differences among the glycerine, methylcellulose, and lidocaine-based groups and the distilled water group (P>0.05). Furthermore, neither Score 2 (CaOH in more than half of the cavity) nor Score 3 (completely filled with CaOH) was observed in the NMP group.

## Discussion

CaOH is a commonly used intracanal medicament in clinical endodontics. The medicament combined with an aqueous vehicle can quickly permeate with the direct contact of the tissue fluids and dissolve away from the root canal (3). However, due to their molecular weight, viscous vehicles dissolve more slowly than aqueous vehicles (4). In addition, the residue of CaOH prevents the penetration of sealers into the dentinal tubules and negatively affects hermetic sealing (12). To date, the removal efficiency of CaOH combined with different vehicles has been investigated using different irrigants and removal techniques (13, 15). However, there is limited data in the literature related to the removal efficacy of NMP-based CaOH medicament from the root canal. Therefore, the purpose of this *in-vitro* ^study was to assess the removal efficacy of CaOH medicament combined with a new NMP agent and other different vehicles from the artificially created grooves in the root canal. According to the present study results, the NMP-based group exhibited better removal efficacy than the CaOH in distilled water. However, the methylcellulose, lidocaine, and glycerine based groups showed cleaning success similar to the distilled water group. Furthermore, Scores 2 and 3 were not observed in the NMP group. Therefore, the null hypothesis was rejected. Similar to our finding, Lim et al. (17) reported that the NMP-based paste showed a smaller percentage of residual volume than the glycerine and propylene glycol groups. Moreover, they detected fewer solid ingredients and precipitate formation in the NMP solution. Therefore, the successful removal efficacy and fewer residual remnants observed in the studies can be attributed to the NMP mechanism increasing the dissolution.^

The mechanism of NMP’s increased solubility is believed to be due to its non-polar carbon structure, which weakens the hydrogen-bound structure of water, allowing it to function as a co-solvent (18). However, the presence of a planar non-polar region causes hydrophobic interactions between NMP and the drug molecule to form a complex. Thus, when the substance dissolves in the NMP, the concentration will increase, and the passive diffusion rate will be faster (19). Accordingly, the increased concentration in the NMP-based solution may have resulted in the medicament diffusing more into the dentinal tubules and less residue in the root canal. These mechanisms can explain the increased removal of NMP compared to the other vehicles in the present study.

Regarding the other finding of this study, glycerine, lidocaine, and methylcellulose-based vehicles have a similar removal efficiency as the distilled water group. However, there have been conflicting results in the literature on this subject. A premixed CaOH paste is a colloid that includes a CaOH powder, liquid (vehicle), and other components to increase the physicochemical properties (3). The percentage of CaOH powder in the manufactured products can differ to achieve optimum consistency, conductivity, and pH, depending on the type of vehicle used. Although the amount of CaOH powder in the medicament can affect the antimicrobial activity or ionic dispersion (19), it was reported that ıt did not affect the removal activity, but the vehicle did (20). Thus, different CaOH powder ratios in the products were not thought to affect removal efficiency for this study.

It is known that a viscous type of vehicle, such as glycerine, is more difficult to remove from the root canals than aqueous vehicles due to the physicochemical properties, internal friction, and high molecular weight (6). Although glycerine has a high molecular weight and a jelly form, it is also a moisturizing agent that can keep medicine smooth (21). Therefore, it should be considered that the setting ability of medicament in a humid environment and body temperatures according to the type of vehicle used. Methylcellulose-based paste (Apexcal) has been shown to have varying degrees of setting in 48 hours, although it is claimed to be a non-setting paste (22). Considering this informations, the similar removal efficiency of water-based medicament and viscous-based medicament obtained in the current study can be attributed to the fact that glycerin in the viscous-based medicament keeps the paste moist. It was also shown in a study using confocal laser scanning microscopy, the penetration into dentinal tubules in the apical region of CaOH pastes prepared with aqueous (distilled water) and viscous (propylene glycol) vehicles were similar (23). They attributed this to the fact that the small diameter, sclerotic, small and fewer tubules and difficulty to reach for the tip of the agitation instruments at the apical region. In this study, the artificial groove was prepared in the apical region.

The conflicting results between the studies may be due to the differences in filling patency, irrigation solutions, and irrigation agitation techniques. Moreover, the results may differ depending on methodologic variations, evaluation techniques, the anatomy of the samples, and the presence of a smear layer.

This study was designed with a standardised size and location of the groove model using a scoring system. The standardised grooves create standardised conditions to provide reliable information about the amounts of medicament before the irrigation (23). Moreover, the design provides an advantage concerning the evaluation, with high-quality intra-examiner reproducibility and good inter-examiner agreement (24). However, it cannot completely mimic the complexity of a natural root canal system. Observer bias in the scoring system is another limitation. Verifying reproducibility using kappa statistics to validate the subjective finding and checking inter-examiner biases are necessary for controlling the resulting quality (25). Therefore, inter-observer reproducibility was reinforced in the current study using a weighted coefficient kappa (Kw). In this study, stereomicroscope evaluation was used, which has the disadvantage of allowing only a two-dimensional assessment that does not give data about the thickness of residual medicament (26). Three-dimensional evaluation can be more helpful in providing more accurate results. In this study, some specimens had no residue of CaOH in the apical region. However, the results may differ on the coronal third of the tooth. It was reported that passive ultrasonic irrigation is more effective in removing CaOH from a standard groove in the apical region than the coronal third of the root canal (27). The mechanism is based on acoustic microcurrent generated in the tip region rather than the coronal tip of the instrument (28). In clinical conditions, the diameter and curvature of roots can also influence the efficacy of irrigation approaches (29). In narrow and curved root canals, the flow of irrigation becomes more difficult, reducing flushing effectiveness (30). Therefore, that study's narrow and curved root canals may generate different results than those observed in this study. Further studies are needed to confirm the findings of the NMP-based materials within the *in-vivo* setup.

## Conclusion

Within the limitations of the present study, the NMP-based CaOH medicament exhibited better removal efficacy than the control group. However, the methylcellulose-, lidocaine-, and glycerine-based groups showed a similar removal efficacy as the distilled water group.

### Disclosures

**Conflict of interest:** The authors deny any conflict of interest.

**Ethics Committee Approval:** This study was approved by the Ethics Committee of Pacific Dental College & Hospital (Date: 08/06/2020, Number: PDCH/20/EC-235).

**Peer-review:** Externally peer-reviewed.

**Financial Disclosure:** This study did not receive any financial support.

**Authorship contributions:** Concept – A.A., B.A., H.A.; Design – A.A., B.A., H.A.; Supervision – A.A., B.A., H.A.; Funding - A.A.; Materials - A.A.; Data collection &/or processing – H.A., A.A.; Analysis and/or interpretation – H.A., B.A.; Literature search – B.A.; Writing – B.A., H.A.; Critical Review – H.A.
